# A Comparative Genomic Study of Attenuated and Virulent Strains of *Babesia bigemina*

**DOI:** 10.3390/pathogens10030318

**Published:** 2021-03-08

**Authors:** Bernardo Sachman-Ruiz, Luis Lozano, José J. Lira, Grecia Martínez, Carmen Rojas, J. Antonio Álvarez, Julio V. Figueroa

**Affiliations:** 1 CENID-Salud Animal e Inocuidad, Instituto Nacional de Investigaciones Forestales Agrícolas y Pecuarias, Jiutepec, Morelos 62550, Mexico; sachman.bernardo@inifap.gob.mx (B.S.-R.); lira.juan@inifap.gob.mx (J.J.L.); martinez.grecia@inifap.gob.mx (G.M.); rojas.carmen@inifap.gob.mx (C.R.); alvarez.jesus@inifap.gob.mx (J.A.Á.); 2 Centro de Ciencias Genómicas, Universidad Nacional Autónoma de México, AP565-A Cuernavaca, Morelos 62210, Mexico; llozano@ccg.unam.mx

**Keywords:** *Babesia bigemina*, attenuated strain, putative virulence genes

## Abstract

Cattle babesiosis is a socio-economically important tick-borne disease caused by Apicomplexa protozoa of the genus *Babesia* that are obligate intraerythrocytic parasites. The pathogenicity of *Babesia* parasites for cattle is determined by the interaction with the host immune system and the presence of the parasite’s virulence genes. A *Babesia bigemina* strain that has been maintained under a microaerophilic stationary phase in in vitro culture conditions for several years in the laboratory lost virulence for the bovine host and the capacity for being transmitted by the tick vector. In this study, we compared the virulome of the in vitro culture attenuated *Babesia bigemina* strain (S) and the virulent tick transmitted parental Mexican *B. bigemina* strain (M). Preliminary results obtained by using the Basic Local Alignment Search Tool (BLAST) showed that out of 27 virulence genes described and analyzed in the *B. bigemina* virulent tick transmitted strain, only five were fully identified in the attenuated laboratory strain. In all cases, the identity and coverture of the identified genes of the wildtype strain were higher than those of the laboratory strain. This finding is putatively associated with the continuous partial loss of virulence genes in the laboratory strain after several passages of the parasite population under optimal in vitro growth conditions. The loss of virulence factors might be reflected in the absence of symptoms of the disease in cattle inoculated with the attenuated strain despite the presence of infection in the bovine host cells.

## 1. Introduction

Bovine babesiosis is caused by *Babesia bovis* and *Babesia bigemina* in Mexico. The disease is distributed in tropical areas and is considered to be one of the most important diseases transmitted by *Rhipicephalus microplus* and *R. annulatus* ticks [1]. The presentation of bovine babesiosis caused by *B. bigemina* is 12–16 days after the ticks feed on the bovine, coinciding with the presence of the intraerythrocytic parasite in the peripheral blood. The temperature rises parallel to the increase in parasitemia to 41–42 °C in two or three days. The infected animals are lethargic, anorexic and have shaggy fur. Hemoglobinemia and hemoglobinuria occur followed by pale mucous membranes along with other signs that may include constipation, dehydration, muscle tremors, weakness, prostration and, if inadequate treatment is instituted, death [2].

*B. bigemina* is an obligate intraerythrocytic Apicomplexan parasite; it belongs to the Protista kingdom, Protozoan sub-kingdom, Apicomplexa phylum, Sporozoasida class, Piroplasmia sub-class, Eucocciiorida order, Piroplasmorina sub-order and Babesiidae family [3]. Parasites have the characteristics of a normal eukaryotic cell with an endoplasmic reticulum, Golgi apparatus, nucleus, microtubules and organelles specific to the Apicomplexa phylum [4]. *Babesia* is a protozoan with alternating (sexual-asexual) reproduction that is characterized by the presence of an apical complex, although incomplete, without a cone but with rhoptries, a polar ring and with sub-pellicular microtubules, micronemes and dense granules known in *Babesia* as spherical bodies. Its shape can be round, piriform or amoeboid [1]. Regarding their biology, they are obligate heteroxenous parasites developing in the invertebrate host (definitive host where the sexual phases of the cycle take place) gametogony and sporogony and in the vertebrate (intermediate) host, binary asexual divisions develop giving rise to merozoites. Various studies have been generated on the bovine host, the agent and their relationships trying to recognize its biological characteristics as well as its distribution to establish programs for the prevention and/or control of the disease. These activities have required the replication of the biological cycle using the parasite-host-vector complex and after multiple tests the artificial growth of this type of parasite has been achieved under in vitro conditions [5,6]. Various methods have been reported to decrease the virulence of pathogenic species in cattle such as rapid passages by parenteral inoculation in splenectomized calves [7], slow passages by parenteral inoculation in intact calves [8], the use of radiation on infected blood [9] and the in vitro culture methodology for the *Babesia* species that affect cattle [5,6,10,11]. The attenuation of intracellular pathogens by in vitro culture is a well known phenomenon and pathogens of viral, rickettsial and protozoal origin (i.e., *B. bovis* and *B. divergens*) have become avirulent or less pathogenic by prolonged growth in cell culture [12,13,14,15]. Work from our research group has shown that a strain of *B. bigemina* maintained by continuous passages in in vitro culture has behaved as an attenuated population as it did not significantly affect the hematological values in inoculated animals [16,17]. The attenuated strain of *B. bigemina* has induced protection from a heterologous challenge with infected blood or infected ticks under controlled and field conditions [16,17,18]. Animals vaccinated with attenuated strains of *Babesia bovis* have been reported to remain as healthy carriers, leading to the emergence of clinical cases due to the reversion to virulence of the strains when they are passed through intact animals or when transmitted by ticks [19]. Vaccine strains have been developed to confer adequate protection without being transmitted by ticks, a trait considered ecologically desirable because it prevents the appearance of clinical cases due to the transmission of the vaccine strain by ticks [20]. This ability has been demonstrated after one passage in susceptible cattle [16,19,20,21]. Furthermore, this type of live attenuated vaccine has the great advantage conferred by its in vitro production: a low risk of contamination with other infectious agents [22,23,24], a highly parasitized suspension of red blood cells and the possibility of relatively large scale vaccine production [25]. Importantly, it was shown that the attenuated *Babesia bigemina* S strain was not transmitted by ticks after several passages in susceptible cattle [26]. The absence of a reversion to virulence of the attenuated *B. bigemina* S strain was demonstrated after three successive passages in susceptible cattle and it was confirmed that the parasites maintained in the in vitro culture lost the ability to multiply in the vector tick during a second pass by sub-inoculation in susceptible cattle [26]. This was evidenced by the absence of kinetes in the hemolymph of ticks fed in cattle with patent parasitemia. Furthermore, it has been shown that even when subjected to three successive passages in highly susceptible cattle, the parasite population maintains its biological characteristic of reduced virulence manifested by the clinical values determined in the sub-inoculated cattle, which did not show relevant changes. As a sexual phase of *Babesia* is required for the infection of the epithelial cells of the midgut of the vector tick [27] and consequently the trans-ovarian transmission [28,29], the parasite population of the Mexican attenuated strain is apparently unable to reproduce sexually in *R. microplus* infested cattle to induce the formation of infective forms for the tick vector. On the other hand, it was not known until now what the components of the parasite responsible for the induction of hemolysis and other pathophysiological effects were in infected cattle.

It is evident that additional studies and research are required using a new generation of sequencing methodologies to identify the possible mechanisms involved in the determination of virulence for the development of the parasite within the erythrocyte infected (and thus cause disease in an inoculated bovine) as well as for the development of subsequent stages of *Babesia* necessary for the infection of the midgut of *R. microplus* (sexual forms) that allow the parasite to acquire again the infective capacity toward the tick vector and, consequently, be trans-ovarially transmitted. Only by using innovative methods of massive sequencing and analysis of the genome and transcriptome shall it be possible to test the hypothesis that a few genetic changes (chromosomal rearrangements, deletions or insertions in genes) occurred in the genome of the attenuated strain that is the subject of study and maintained in an in vitro culture, which are reflected in an attenuated phenotype lacking the ability to be transmitted by the tick vector *R. microplus*. In this study we performed a bioinformatic analysis of possible plasticity on the genome of *B. bigemina* by sequencing the genome of a virulent strain and an attenuated strain (derived from the virulent isolate but maintained in continuous in vitro cultivation in Mexico). The list of genes associated with the virulence of *B. bigemina* is called the virulome of *B. bigemina*.

## 2. Results and Discussion

The genome sequencing and assembly analysis elucidated that the attenuated *Babesia bigemina* laboratory strain contained a 9,180,241 pb and a wildtype 11,852,459 pb, which represented a 22.545% difference (Table 1). Interestingly, the guanine-cytosine (GC) content was slightly higher in the laboratory strain of *B. bigemina* (52.3%) versus the wildtype (50.74%).

**Figure 1:**
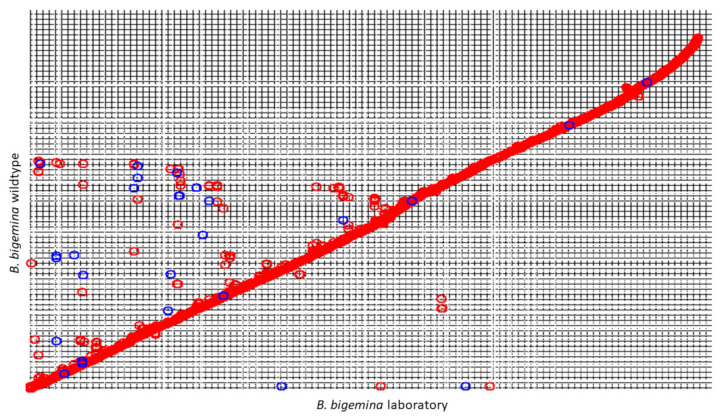
Dot plot of the genomic synteny comparison between *B. bigemina* wildtype versus laboratory strains. The circles in red represent the genes that keep the wildtype (5’–3’) orientation and the blue ones the genes that are in a position opposite to the wild strain.

Although nucleotide variations in the sequencing effort were important, the synteny was maintained as can be observed in the comparative dot plot in Figure 1.

### Genomic Gene Virulence Differences between Babesia bigemina Strains

The virulence gene sequences were obtained from a Basic Local Alignment Search Tool (BLAST) search and the differences between the strains were remarkable; while the wildtype strain presented 27 genes with a coverture average of 98.06%, the attenuated laboratory strain only presented five of those genes with 81.2% of coverture (Table 2). The most common type of genes identified in both strains were putative partial genes; 18 for the virulent wildtype and three for the attenuated strain. The only identified hypothetical gene belonged to the wildtype strain with the shortest sequence observed and zero mismatches (107 bp; Table 2). The largest gene (6117 bp) present in the virulent strain had the highest number of mismatches (216 bp; Table 2). Attempts were made to predict whether those genes would translate into proteins. For the predictions of the eukaryote genes, we usually utilize the Augustus online program, which predicts in the six open reading frames the possible forms of proteins. Unfortunately for us, the genome assembly performed with the sequence reads still contained too many contigs, which made an adequate bioinformatic analysis not possible. This was solved by performing high-throughput genome sequencing on a PacBio Single Molecule Real Time (SMRT) sequencing platform to close gaps or extend contig ends in the Illumina assembly [30].

In addition, considering that the phenotype differences among the culture attenuated and the wildtype strain included a lack of transmission of the cultured strain by ticks, a comparative analysis of gene encoding for proteins involved in the sexual stage development, kinete-specific or other genes required for tick transmission was performed. Several genes that are involved in the parasite development in the tick vector are well described in the literature such as the hap2 and CCp gene family (Table 3) [31,32,33,34,35]. It was found that these genes were well conserved in both genomes analyzed with a slightly higher sequence identity in the attenuated strain. Interestingly, CCp2 and BBBOND genes were identified in two different contigs and apparently a few nucleotides were missing (406 bp and 61 bp, respectively). The CCp1 gene described in *B. bovis* [33] was apparently missing in both the virulent and the attenuated strain genomes (Table 3). Regarding the developmental genes described in Table 3 that seemed to have a similar homology no matter whether they belonged to the avirulent isolate or the virulent one, previous studies have shown that the CCp gene family members of *Babesia* parasites are differentially expressed throughout the life cycle and data presented in those studies demonstrated that the *Babesia* CCcp genes are predominantly expressed during parasite replication in the tick vector [32,33,34,35]. Whether this is the case for the CCp genes identified in this comparative genomic study needs further investigation and we are currently preparing the appropriate in vivo and in vitro experiments to test that by RT-PCR.

The genome sequencing effort was performed with two replicates for each parasite strain. The genome assembly was achieved with two sets of reads for each strain and these data are presented in Table 1. The BLAST analysis searching for the genes of interest was performed with both contigs of the assembly obtained with the reads from the two technical replicates for both *Babesia* strains and separately with contigs from the reads of the two technical replicates of each *Babesia* strain. Despite having shorter contigs in the individual technical replicates, the results of the analysis obtained were essentially the same as those presented in Table 2 and Table 3 when the replicates were submitted to BLAST analysis.

Genetic changes have been identified in the genome of other Apicomplexa agents cultured in vitro [36,37]. In addition, the comparative analysis of the genome of the Mexican virulent strain with the genome of the Australian virulent strain called Bond [38] allowed the mapping of virulent gene sequences against a reference genome (Australian *B. bigemina* genome) as performed with BLAST. This approach allowed a first analysis of the genome composition of each strain and identified possible changes (deletions or insertions) or chromosomal rearrangements that occurred in the genomes of the virulent and attenuated *B. bigemina* strains. For a pathogen with a relatively small genome such as that representing *Babesia*, which for the case of the Bond strain is estimated to have 4457 genes, the completion of the genome sequences will provide the complete genetic repertoire of antigens and virulence factors from which new candidates as targets for treatment or immune prophylaxis can be identified. It has been estimated that more candidates can be identified (between 10 and 100 times more) using genomics-based approaches than those identified through conventional methods in the same period. Research studies implemented on the development of genomic-based vaccines have substantively increased our understanding of the physiology, epidemiology, pathogenicity and protein function of pathogenic organisms [39,40,41]. Traditionally, sequencing projects have focused on the genome study of pathogenic and virulent organisms. A comparison of genomes of pathogenic and non-pathogenic strains allows the identification of targets for the development of vaccines and therapeutic drugs based on proteins that are specifically involved in pathogenesis and virulence. Comparative genomics allows the identification of unique genes from certain pathogenic strains that may be absent in apathogenic strains. This comparative filter significantly decreases the multitude of potential targets to be sifted [38].

Recent advances in the development of in depth sequencing technologies open new possibilities for a variety of applications in research at the transcriptional level of an organism. The information provided by genome-level transcription studies is important for understanding the biology of an organism in the context of a system and particularly when the interface with the entire genome sequence is performed, enabling the analysis of gene transcription in relation to the organization of the genome [41].

Gene loss in other eukaryotic parasites of several animals that appear to have adapted to live in blood spaces and even an obligate intracellular lifestyle by modifying the morphology and content of their cells has also been observed [42,43]. Gene loss or reductive evolution is so extensive that it has been suggested to be the dominant mode of biological evolution in complex organisms [44].

In this study we present an evolutionary reduction due to the apparent loss of genes for the first time reported in *B. bigemina* observed in a strain that has been maintained for several years under laboratory conditions, which has lost more than 80% of the analyzed virulence genes compared with the wildtype strain. Alternatively, whether these changes were due merely to the selection of a parasite sub-population that previously existed in the original strain that underwent culturing needs to be experimentally tested. Undoubtedly, this also has to be corroborated by transcriptional analysis in future research studies. It will be interesting to analyze the repertoire of differentially expressed genes in the attenuated *B. bigemina* strain and transcriptome studies are on course to determine if the differential gene expression is maintained [45] or if it varies with respect to the wildtype strain.

## 3. Materials and Methods

### 3.1. Parasites

The attenuated strain of *B. bigemina* is a population of parasites originally derived from a virulent isolate from Mexico collected from a clinical case of babesiosis. The strain was adapted to an in vitro culture using a microaerophilic stationary system [6]. Once established in in vitro cultivation it was continuously cultivated for almost five consecutive years in the laboratory with passes every 72 or 96 hrs (at least 500 sub-cultures in cultivation). Thereafter, the in vitro culture of the *B. bigemina* was carried out discontinuously and with an indefinite number of passes in cultivation; it was grown for short periods of time (one or two months), just until enough material was obtained for experiments that were conducted during the development of the attenuated live vaccine (periods 1991–1998 and 2001–2006) and immediately frozen down in liquid nitrogen until other experiments were conducted in the period 2011–2017 in which it was cultivated for the latest experiments (tick transmissibility) and immunogenicity validation [17,18,23,24,26] until now when sequencing was carried out. More importantly to note is that the attenuated biological material has not been passed through a bovine host since its original isolation unlike the virulent isolate (from which the attenuate strain was selected) and that is replicated in animals whenever required. Morphologically, the attenuated strain has not changed; it keeps infecting erythrocytes both in vivo and in vitro but it does not cause disease when inoculated in cattle and has been maintained by alternating continuous cultivation and cryopreservation since then [17,18,23,24,26]. The virulent strain, originally isolated from a field clinical case, has been maintained through tick passages in susceptible animals and cryopreservation in liquid nitrogen [6,17,26].

### 3.2. Genomic DNA Extraction

Bovine erythrocytes infected with the different populations of *B. bigemina* were used to carry out the extraction of genomic DNA by conventional methods [46]. Between 20 and 30 µg of genomic DNA was used to prepare the two clonal libraries required for each strain.

### 3.3. Genome Sequencing of B. bigemina, Attenuated and Virulent Strains

Whole genome sequencing was performed at the next-generation sequencing Illumina Mi-Seq (Illumina, San Diego, CA, USA) core facility of the National Autonomous University of Mexico located at the Biotechnology Institute in Cuernavaca, Morelos. The sequence readings obtained with the Illumina system were assembled de novo using the SPAdes program (version 3.1.1) (Algorithmic Biology Laboratory, Saint Petersburg Academic University, St. Petersburg, Russia) [47]. The contigs generated were ordered with the MUmmer tool [48]. The sequencing effort obtained 264× and 89× of coverage (attenuated and virulent strains, respectively) to obtain the genome sequences. The list of selected genes was based initially by considering previous articles on classical immunology and transcriptomic studies where it was mentioned that those were genes attributed to virulence or potential virulence factors [32,49]. The mapping of virulent gene sequences against a reference genome (Australian *B. bigemina* genome) was performed with BLAST [50]. This allowed a first analysis of the genome composition for each strain and identified possible changes (deletions or insertions) or chromosomal rearrangements that occurred in the genomes of the virulent and attenuated *B. bigemina* strains.

### 3.4. Data Accession

This Whole Genome Shotgun project has been deposited in GenBank under the accession Genome number JAFBJA000000000, BioSample accession SAMN17098652, BioProject ID PRJNA685856 for the attenuated strain and Genome submission SUB8750815, BioSample accession SAMN17098654, BioProject ID PRJNA685857 for the virulent strain.

## Figures and Tables

**Table 1:** Sequencing effort of the *Babesia bigemina* strains analyzed in this work.

Sequencing Data *\ Strain	*B. bigemina* Attenuated	*B. bigemina* Virulent
**# Contigs**	3914	1537
**Total length**	9,180,241	11,852,459
**GC (%)**	52.3	50.74
**Coverage (X)**	264	89
**N50**	4599	41,154
**N75**	1682	7155
**L50**	463	85
**L75**	1318	230
**# N’s per 100 kbp**	0.03	0.01
**GenBank accession no.**	JAFBJA000000000	PRJNA685857

* Statistics are based on contigs of size > = 500 bp. # = Number of contigs. GC = Guanine-Cytosine content. #N’s = Number of unasigned Nucleotides.

**Table 2:** Virulence genes of *B. bigemina* wildtype and laboratory strains.

Name	Gene	Accession no.	Contig_number	% Identity *	Alignment Length	Mismatches	Query Start	Query End	E-value
Virulent Strain									
Calcium-dependent protein kinase 4	PP **	XM_012911530.1	contig00169	99.08	1518	14	1	1518	0
Calmodulin-domain protein kinase 2	PP	XM_012910710.1	contig00134	98.75	2322	29	1	2322	0
cAMP-dependent protein kinase	PP	XM_012914446.1	contig00026	98.88	1068	12	1	1068	0
Casein kinase I	PP	XM_012913338.1	contig00174	98.62	1017	14	1	1017	0
cGMP dependent protein kinase	cGMP	XM_012914849.1	contig00056	99.21	1644	13	1	1644	0
Cyclin 4	PP	XM_012914402.1	contig00018	98.62	506	7	638	1143	0
Diphosphate kinase family, putative	PP	XM_012913759.1	contig00012	99.34	302	2	1	302	1 × 10^−166^
dnaJ C terminal region domain	dnaJ	XM_012910446.1	contig00220	98.74	1194	15	1	1194	0
Glycerol kinase	PP	XM_012914443.1	contig00026	98.99	296	3	1030	1325	4 × 10^−160^
Glycogen synthase kinase-3 alpha	PP	XM_012910925.1	contig00076	98.86	264	3	174	437	4 × 10^−141^
Hypothetical gene	HG ***	XM_012911455.1	contig00674	100	107	0	1316	1422	2 × 10^−54^
Merozoite surface glycoprotein	gp45	AF298630.1	contig00098	92.31	286	21	919	1204	2 × 10^−106^
Mitogen-activated protein kinase	PP	XM_012912499.1	contig00013	98.23	1803	32	1	1803	0
Phosphatidylinositol 3-and 4-kinase	PP	XM_012912148.1	contig00156	97.66	3417	80	1	3417	0
Phosphatidylinositol 4 kinase	PP	XM_012911128.1	contig00059	97.19	3417	96	1	3417	0
Phosphatidylinositol-4-phosphate 5-kinase	PP	XM_012914497.1	contig00063	99.1	334	3	855	1188	0
Phosphatidylinositol-4-phosphate 5-kinase	PP	XM_012910512.1	contig00138	96.47	6117	216	1	6117	0
Probable fructokinase	PP	XM_012910446.1	contig00220	98.74	1194	15	1	1194	0
Protein kinase domain	kinD	XM_012914171.1	contig00006	98.32	2082	35	1	2082	0
Putative rhoptry protein	rap-1c	NC_027216.1	contig00167	96.66	1529	51	1	1529	0
RAP-1 related antigen	rra	NC_027216.1	contig00003	97.46	1062	27	1	1062	0
Related serine/threonine protein kinase	MAPKK	XM_012911485.1	contig00028	95.7	744	32	538	1281	0
Ser/Thr protein kinase	PP	XM_012910695.1	contig00064	96.96	723	22	340	1062	0
Serine/threonine kinase	PP	XM_012914264.1	contig00171	98.78	1893	23	1	1893	0
Serine/threonine kinase 1	PP	XM_012914521.1	contig00041	98.23	960	17	1	960	0
Transcription factor TFIIB	TFIIB	XM_012912762.1	contig00016	98.16	1575	29	1	1575	0
Transcription initiation factor TFIIB	PP	XM_012910684.1	contig00064	98.67	975	13	1	975	0
Attenuated Strain									
cGMP dependent protein kinase	cGMP	XM_012914849.1	contig00032	84.55	110	17	1795	1904	1 × 10^−15^
Calcium-dependent protein kinase 4	PP	XM_012911530.1	contig00066	78.68	469	100	320	788	6 × 10^−32^
Phosphatidylinositol 4 kinase	PP	XM_012911128.1	contig00009	80.52	426	83	2968	3393	2 × 10^−46^
Serine/threonine kinase	PP	XM_012914264.1	contig00035	83.54	164	27	1111	1274	4 × 10^−24^
Transcription factor TFIIB	TFIIB	XM_012912762.1	contig00023	78.73	569	121	37	605	2 × 10^−41^

* Percent identity compared with the reference genome (Australian *B. bigemina* BOND genome). ** PP, putative partial. *** HG, hypothetical gene.

**Table 3:** Development genes of *B. bigemina* wildtype and laboratory strains.

Name	Gene	Accession No.	Contig_number	% Identity *	Alignment Length	Mismatches	Query Start	Query End	E-value
Virulent Strain	hap2	NC_027218.1:c1816274-1813816	NODE_36_length_66145_cov_3.064019	96.928	2474	61	1	2459	0
	BbiKSP	NC_027217.1:1488918-1490756	NODE_115_length_32231_cov_1.736940	97.879	1839	39	1	1839	0
	CCp1	XM_001611715.1 **	Not Found						
	CCp2	NC_027217.1:905825-910977	NODE_16_length_99920_cov_2.450711	97.846	5153	105	1	5153	0
	CCp3	NC_027218.1:c3038315-3033906	NODE_11_length_111305_cov_2.393297	97.937	4410	91	1	4410	0
	BBBOND_0204030	NC_027217.1:c927096-926018	NODE_16_length_99920_cov_2.450711	96.762	1081	33	1	1079	0
Attenuated Strain	hap2	NC_027218.1:c1816274-1813816	NODE_28_length_21819_cov_0.316719	96.888	2474	62	1	2459	0
	BbiKSP	NC_027217.1:1488918-1490756	NODE_840_length_2631_cov_0.246875	97.662	1839	43	1	1839	0
	CCp1	XM_001611715.1 **	Not Found						
	CCp2	NC_027217.1:905825-910977	NODE_145_length_10242_cov_0.314817	98.031	2895	51	1	2895	0
		NC_027217.1:905825-910977	NODE_328_length_6126_cov_0.306193	97.841	1853	40	3301	5153	0
	CCp3	NC_027218.1:c3038315-3033906	NODE_133_length_10700_cov_0.285351	98.05	4410	86	1	4410	0
	BBBOND_0204030	NC_027217.1:c927096-926018	NODE_281_length_6848_cov_0.245979	96.44	899	30	1	897	0
		NC_027217.1:c927096-926018	NODE_1850_length_1188_cov_0.253357	99.18	122	1	958	1079	3 × 10^−57^

* Percent identity compared with the reference *B. bigemina* genes. ** Sequence from *Babesia bovis.*

## Data Availability

The data presented in this study are available on request from the corresponding author.
